# The Physiological Functions and Therapeutic Potential of Hypoxia-Inducible Factor-1α in Vascular Calcification

**DOI:** 10.3390/biom14121592

**Published:** 2024-12-12

**Authors:** Zhenghong Zhang, Defan Wang, Renfeng Xu, Xiang Li, Zhengchao Wang, Yang Zhang

**Affiliations:** 1 Provincial Key Laboratory for Developmental Biology and Neurosciences, College of Life Sciences, Fujian Normal University, Fuzhou 350007, China; zhangzh@fjnu.edu.cn (Z.Z.); xurenfeng131772@126.com (R.X.); 2 Fujian Provincial Key Laboratory of Reproductive Health Research, School of Medicine, Xiamen University, Xiamen 361102, China; defanwang@126.com; 3 Department of Pharmacological and Pharmaceutical Sciences, College of Pharmacy, University of Houston, Houston, TX 77204, USA; xli61@central.uh.edu

**Keywords:** hypoxia-inducible factor-1α, vascular smooth muscle cell, energy metabolism, vascular calcification, therapeutic target

## Abstract

HIF-1α plays a crucial regulatory role in vascular calcification (VC), primarily influencing the osteogenic differentiation of VSMCs through oxygen-sensing mechanisms. Under hypoxic conditions, the stability of HIF-1α increases, avoiding PHD and VHL protein-mediated degradation, which promotes its accumulation in cells and then activates gene expressions related to calcification. Additionally, HIF-1α modulates the metabolic state of VSMCs by regulating the pathways that govern the switch between glycolysis and oxidative phosphorylation, thereby further advancing the calcification process. The interaction between HIF-1α and other signaling pathways, such as nuclear factor-κB, Notch, and Wnt/β-catenin, creates a complex regulatory network that serves as a critical driving force in VC. Therefore, a deeper understanding of the role and regulatory mechanism of the HIF-1α signaling during the development and progression of VC is of great significance, as it is not only a key molecular marker for understanding the pathological mechanisms of VC but also represents a promising target for future anti-calcification therapies.

## 1. Introduction

Vascular calcification (VC) is a significant pathological feature in cardiovascular diseases, characterized by the deposition of calcium salts in the vascular wall [1,2]. This process is strongly associated with various cardiovascular conditions, particularly in patients with CKD, hypertension, and diabetes [3,4]. Studies related to CKD have shown that cardiovascular calcification is more advanced and severe in patients with kidney disease than in non-CKD individuals. This difference may be related to the impact of CKD on calcium and phosphorus metabolism, leading to increased deposition of calcium salts in blood vessels [5]. VC is highly prevalent in patients with CKD and is significantly associated with the incidence and mortality rates of cardiovascular diseases [6]. Cardiovascular complications in patients with CKD often lead to increased mortality, highlighting the clinical importance of VC [6,7,8].

Hypertension-related studies have shown that coronary artery calcification is associated with the development of hypertension and may play a critical role in the progression of this condition [9]. There is a significant interaction between hypertension and arterial stiffness, which is strongly associated with VC [10]. In addition, VC not only affects the mechanical properties of blood vessels but may also have a profound impact on the occurrence and progression of hypertension [11], exacerbating the health risks associated with this condition [12]. Studies on diabetes indicate that the incidence of VC in diabetic patients is significantly higher than in non-diabetic individuals. The VC caused by diabetes is a key factor contributing to vascular sclerosis and dysfunction, which constitutes a significant health risk for patients [13,14]. In addition, research on the implications of VC in the brain vasculature suggested that VC can lead to increased stiffness and decreased elasticity of the vascular walls, thereby significantly affecting the regulation of cerebral hemodynamics [15,16]. It is an important risk factor for cerebrovascular disease, mainly including an increased risk of stroke, impaired self-regulation of cerebral blood flow, and cognitive dysfunction [15,16].

VC, as an active and regulated process, is intimately linked to cardiovascular disease. It is not merely a result of arteriosclerosis but is also strongly associated with pathological cardiovascular conditions, including hypertension [17]. Ni et al. found that elevated serum calcium levels are positively correlated with VC and cardiovascular disease risk, highlighting the necessity of monitoring and managing serum calcium levels to prevent cardiovascular disease [18]. Rubin and Lian et al. demonstrated the strong association between VC and conditions like atherosclerosis, myocardial ischemia, and heart failure, underscoring the critical need for monitoring vascular calcification [19,20]. Collectively, VC is widely acknowledged as a critical predictor of cardiovascular disease and plays a pivotal role in influencing clinical outcomes [2].

In recent years, studies have demonstrated that HIF-1α serves as a critical regulator of VC [3,4,21]. Negri et al. reported that HIF-1α mRNA and protein expressions were significantly elevated in calcified vascular cells compared to non-calcified cells, suggesting that HIF-1α is essential in VC [22]. Further research has shown that HIF-1 plays a crucial role in phosphate-induced calcification of VSMCs, and the oxygen-dependent degradation of HIF-1α mediated by PHDs is implicated in the progression of VC [22]. Li et al. found that elevated serum HIF-1α levels are associated with VC in patients with type 2 diabetes, indicating that HIF-1α not only contributes to the pathophysiology of diabetes but also to the development of VC [23].

HIF-1α is a transcription factor expressed under hypoxic conditions that governs the expression of a wide range of genes, including those involved in calcium metabolism and inflammatory responses. Weidemann et al. found that HIF-1α modulates the expression of over 100 genes, including those directly or indirectly affecting calcium metabolism. HIF-1α influences cellular energy metabolism and adaptive responses by modulating these gene expressions, thereby promoting calcium transport and storage [24]. Thus, HIF-1α not only directly participates in calcium metabolism regulation but also enhances cellular adaptability to hypoxic environments through other metabolic pathways [25]. Additionally, HIF-1α is crucial for inflammatory response, primarily by driving a metabolic shift toward glycolysis [26]. This metabolic transformation is a critical mechanism enabling cells to adapt to hypoxic environments and is strongly linked to the development of inflammation-related diseases [26]. Kiani et al. demonstrated that inhibition of the HIF-1α gene in myeloid cells reduces ATP production and significantly dampens the inflammatory response, highlighting the interplay between HIF-1α, energy metabolism, and inflammation [27]. Similarly, Imtiyaz et al. found that HIF-1α deficiency in myeloid cells results in markedly reduced levels of pro-inflammatory cytokines, such as TNF-α and IL-1/12, underscoring its pivotal role in regulating inflammatory mediators and influencing the broader immune response [28].

Studies have demonstrated that when HIF-1α levels rise, it enhances the osteogenic phenotype of VSMCs, thereby promoting VC [4,19]. Csiki et al. reported that sustained activation of HIF-1α not only impairs cell proliferative capacity but also induces osteogenic differentiation in valve interstitial cells [29]. Similarly, Negri et al. found that HIF-1α is crucial for the transdifferentiation of VSMCs through upregulation of osteogenic-related genes, providing insights into the mechanisms underlying HIF-1α’s role in VC and associated pathologies [22].

HIF-1α drives the progression of VC through the regulation of multiple mechanisms, including oxygen supply, glucose metabolism, and cell proliferation [1,2,3]. When oxygen demand exceeds supply, HIF-1α stabilizes, increasing its transcriptional activity and activating genes involved in hypoxic adaptation [30]. For example, HIF-1α enhances the expression of energy metabolism-related genes, facilitating cellular survival under hypoxic conditions [31]. HIF-1α activity is regulated by oxygen concentration as well as other factors, including nutrients and metabolites [32]. Shen et al. demonstrated that HIF-1α upregulates glucose transporter 1, significantly increasing glucose uptake and enhancing aerobic glycolysis, enabling cells to maintain energy production under hypoxia [33,34,35]. Similarly, Soucek et al. found that HIF-1α facilitates cellular adaptation to hypoxia and hypoglycemia by upregulating genes involved in erythropoiesis, angiogenesis, and glucose metabolism, highlighting its central role in preserving cellular energy homeostasis [36].

In addition, Infantino et al. found that under hypoxic conditions, HIF-1α enhances rapid cellular proliferation by modulating glycolysis and lactate fermentation [34]. Jiang et al. demonstrated that elevated HIF-1α activates downstream target genes, mitigates hypoxia-induced cellular and organ damage, and supports cell survival, highlighting the dual role of HIF-1α in promoting proliferation and ensuring survival [37]. Interestingly, Carrera et al. found HIF-1α deficiency results in cell cycle arrest, thereby suppressing cell proliferation, emphasizing the critical importance of HIF-1α in cell growth and proliferation [38].

This review aims to provide a comprehensive overview of the role and regulatory mechanisms of HIF-1α during the development of VC and to explore its potential as a therapeutic target in the future.

## 2. HIF-1α Overview

**Figure 1:**
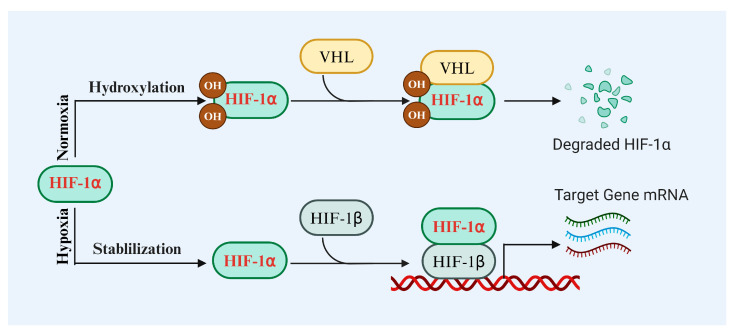
HIF-1α metabolism under normoxic and hypoxic conditions. VHL: the Von Hippel–Lindau ubiquitin-E3 ligase complex.

HIF-1α, a key transcription factor, senses oxygen levels and regulates a range of biological processes [39,40,41,42,43,44]. Ziello and Infantino et al. analyzed the structure of the HIF-1α and HIF-1β genes, finding that HIF-1α/β consist of 826 and 789 amino acids, respectively. Both contain the bHLH motif and PAS domain, which are crucial for their function as transcription factors [34,45]. Under hypoxic conditions, stabilized HIF-1α binds with HIF-1β in the nucleus to form heterodimers. These heterodimers regulate the expression of numerous target genes, fulfilling critical physiological functions [46,47,48,49,50,51,52], such as angiogenesis, glucose metabolism, cell proliferation and survival, as well as invasion and metastasis (Figure 1). Semenza demonstrated that HIF-1α promotes erythropoietin expression, stimulating red blood cell production and enhancing oxygen transport capacity [53]. Zimna and Kurpisz found that HIF-1α promotes VEGF expression, facilitating new blood vessel formation and improving blood supply in hypoxic areas [54]. Taylor and Scholz observed that, in hypoxic environments, HIF-1α drives a shift toward anaerobic metabolism to enhance energy production efficiency [55]. Weidemann and Johnson reported that HIF-1α can inhibit apoptosis, enhance cell tolerance to hypoxia and other stresses, and promote cell survival by upregulating anti-apoptotic genes [24]. Ruan and Chu et al. found that activated HIF-1α supports tumor cell growth by promoting angiogenesis and metabolic changes [56,57]. Ruan and Zimna further demonstrated that HIF-1α activation promotes the production of inflammatory mediators, influencing immune cell function and enhancing the body’s response to pathogens during infection and inflammation [56,58]. Taylor and Weidemann also noted that HIF-1α regulates immune cell metabolism under hypoxic conditions, boosting their activity and function, thereby enhancing the overall immune response [24,55]. Additionally, Zimna and Kurpisz found that HIF-1α participates in bone tissue formation and remodeling by regulating related gene expression [54]. Moreover, Taylor and Scholz highlighted that HIF-1α protects nerve cells during injury and disease by promoting angiogenesis and inhibiting apoptosis [55].

HIF-1α is involved in cellular metabolic adaptation, angiogenesis, and red blood cell production, which are pivotal during the progression of various diseases [46,47,50]. Papandreou and Lee demonstrated that HIF-1α promotes glycolysis by regulating multiple metabolism-related genes [59], facilitating energy production under hypoxic conditions [25]. Zimna and Kurpisz found that HIF-1α transcribes and activates various genes related to angiogenesis, including VEGF, PlGF, PDGFB, ANGPT1, and ANGPT2, which are crucial for the formation of new blood vessels [54]. Lin et al. demonstrated that in hypoxic conditions, HIF-1α expression is significantly elevated, enhancing the transcriptional activity of its downstream genes and promoting angiogenesis [60]. Franke and Garcia et al. demonstrated that under hypoxic or anemic conditions, HIF-1α promotes erythropoiesis by upregulating EPO gene expression and regulates EPO production in both the kidneys and other tissues, including the liver [61,62,63]. HIF-1α also plays a significant role in various diseases that generate hypoxic microenvironments, including cancer, stroke, and heart disease. Particularly under hypoxic conditions in tumors, the expression of HIF-1α activates genes that promote tumor growth, resulting in more invasive tumor phenotypes [64]. Ziello et al. found that overexpression of HIF-1α promotes angiogenesis, thereby enhancing oxygenation within tumor regions [45], besides the role of relevant proteome motifs as Asn-Gly-Arg in angiogenesis [65]. Masoud and Akanji et al. found a strong correlation between elevated levels of HIF-1α and tumor metastasis, angiogenesis, and poor patient prognosis [66]. HIF-1α stimulates cancer progression by promoting cell proliferation and metabolic changes [67,68]. Inhibiting HIF-1α activity can limit cancer progression, and HIF-1α is considered a feasible target for cancer treatment [69]. As research on HIF-1α deepens, the potential use of HIF inhibitors in cancer therapy has gained increasing interest [70]. In addition, HIF-1α promotes excessive ECM production, contributing to persistent fibrosis in various organs [71]. Therefore, understanding the structure and function of HIF is vital for the development of new therapeutic strategies, particularly in the treatment of ischemic diseases and tumors [72].

HIF-1α is crucial in vascular calcification (VC) and its regulation, with its expression and activity modulated by multiple factors [3,22,49,73]. Lim et al. demonstrated that HIF-1α expression was significantly elevated in numerous cardiovascular diseases [74], and this increase is closely associated with coronary artery calcification in diabetic patients [23,75]. VC is a multifaceted process involving an increase in calcification-inducing factors and a decrease in calcification-inhibiting factors. Among these, inorganic pyrophosphate, a key calcification inhibitor, is closely linked to HIF-1α activity, particularly in its synthesis and homeostasis regulation [76]. HIF-1α also contributes to VC regulation by promoting the expression of key enzymes in the glycolysis pathway, thereby altering cellular metabolism [77]. Furthermore, HIF-1α further contributes to VC progression through the regulation of inflammatory factors [77,78]. Therefore, intervention strategies targeting HIF-1α could potentially mitigate or delay VC progression. Exploring the regulatory mechanisms of HIF-1α, particularly its response to hypoxic conditions, may offer valuable insights into the onset and progression of VC [1,20].

## 3. Vascular Calcification

**Figure 2:**
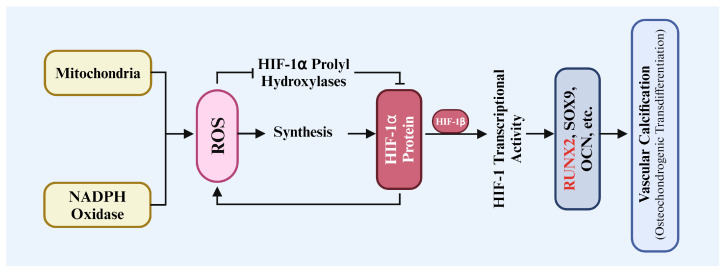
Role of HIF-1α in ROS-triggered osteochondrogenic transdifferentiation of VSMC during vascular calcification.

VC is an abnormal pathological process of biomineralization, characterized by the deposition of mineralized calcium, primarily as hydroxyapatite, within blood vessels [1,4]. VC is an active and regulated process (Figure 2), and it is closely linked to numerous cardiovascular diseases, particularly coronary atherosclerosis and hypertension [2,20,79]. Abedin et al. found that specific clinical conditions, such as diabetes, menopause, and osteoporosis, can exacerbate VC, with these factors interrelated through various mechanisms that further promote VC progression [80]. Raggi and Bellasi found that the risk of cardiovascular calcification markedly increases in the advanced stages of CKD, indicating that VC is not only a marker of disease progression but also a key determinant of poor prognosis [81].

VC can be classified into two main types based on the location of calcification: intimal calcification and medial calcification [82]. Intimal calcification commonly occurs in the intima of arteries, often indicating the presence of peripheral vascular disease, which can restrict blood flow and is frequently associated with atherosclerosis. Intimal calcification is particularly common in elderly individuals and high-risk cardiovascular patients, especially in atherosclerotic plaques, where it may lead to vascular lumen stenosis, thereby affecting blood flow [83]. In contrast, medial calcification primarily occurs in the middle layer of blood vessels. While medial calcification typically does not cause vascular stenosis, it increases blood vessel rigidity, thus affecting blood pressure and hemodynamics, and is often associated with aging, diabetes, and CKD [84]. Cardiovascular calcification was considered a passive degenerative process in which hydroxyapatite accumulates in different places of the cardiovascular system. By now, this paradigm has changed, and it has become broadly accepted that calcification is a markedly regulated process. Additionally, venous calcification shares similarities with arterial calcification, narrowing the vascular lumen, altering blood flow velocity, and potentially increasing turbulence, thereby raising the risk of thrombosis [80,85]. Coronary artery calcification can stiffen the vessel wall, significantly decreasing elasticity and increasing stiffness, which weakens the regulatory function of the coronary artery, impairing its adaptability to changes in blood flow demand [86,87,88]. It is evident that VC not only affects blood vessel elasticity but also leads to severe health complications, including heart disease and stroke [2].

Research has shown that the occurrence of VC is not a simple passive process but rather a complex physiological and pathological process involving various cell types, signaling pathways, and biomolecules [2]. In this process, VSMCs undergo transformation into osteoblast-like cells, promoting calcium deposition and bone matrix formation. A better understanding of the mechanisms underlying VC could offer valuable insights for the development of novel therapeutic strategies, such as slowing down or reversing the calcification process through drug intervention [20,83].

Chronic inflammation, mediated by cytokines such as TNF-α and IL-6 secreted by immune cells, can drive the calcification of smooth muscle cells. Wang and Cai et al. have shown that inflammation is considered an important driving factor for VC, and the release of pro-inflammatory cytokines creates a favorable microenvironment for calcification, leading to functional changes in VSMCs [89,90]. Among these, TNF-α is a major pro-inflammatory cytokine that participates in various inflammatory responses. In the context of VC, TNF-α not only directly stimulates the calcification process of smooth muscle cells but also enhances the calcification response by inducing the release of other pro-inflammatory factors [91,92]. At the same time, TNF-α can reduce the expression of fetuin-A, a natural calcification inhibitor, and its deficiency can exacerbate calcification [93]. IL-6, another crucial pro-inflammatory cytokine, works in conjunction with TNF-α in the inflammatory process. In VC, IL-6 directly promotes the calcification process by promoting smooth muscle cell proliferation and osteogenic differentiation [94]. Moreover, the release of IL-6 can trigger a series of cascade reactions, which, in turn, stimulates the production of additional inflammatory factors, such as IL-1β, further exacerbating calcification [95,96].

Moreover, oxidative stress contributes to cellular damage and accelerates the calcification process. Tóth et al. showed that excessive ROS contribute to the activation of certain osteogenic signaling pathways, thereby promoting calcification of VSMCs [97]. Byon et al. demonstrated that oxidative stress can alter intracellular signaling by regulating the osteogenic transcription factors Runx2 and AKT, thereby enhancing calcium deposition in VSMCs, further promoting VC [98]. Sánchez Milán et al. indicated that oxidative stress can alter cellular signaling such as t-Cys and interact with HIF-1α effects [99].

In pathological conditions such as CKD, an imbalance in calcium and phosphorus metabolism directly contributes to the development of VC. Shanahan et al. showed that metabolic imbalance of calcium and phosphorus is a common phenomenon in patients with CKD and that dietary regulation of calcium and phosphorus intake may mitigate the calcification process [100]. Cozzolino et al. analyzed the central role of phosphorus in VC and pointed out that in patients with CKD, excessive phosphorus levels not only promote calcification formation but may also affect cardiovascular health [101]. Xiong et al. showed that elevated serum calcium levels increase the risk of vascular disease under high phosphate conditions, especially in CKD patients [102].

Moreover, metabolic abnormalities of vitamin D are also strongly linked to calcification [1,2]. Lack or excess of vitamin D can contribute to the development of VC. Drüeke and Massy explored the complex role of vitamin D and its derivatives in VC, highlighting that vitamin D deficiency is linked to increased calcium deposition [103]. Razzaque studied the relationship between vitamin D and VC and found that lower levels of vitamin D were associated with the occurrence of VC in an experimental uremic model. In addition, excessive vitamin D activity has been shown to induce VC, and reducing vitamin D levels can reverse this vascular pathology [104]. Hou et al. investigated the protective effect of vitamin D supplementation in patients with CKD and found that vitamin D can reduce VC in endothelial cells by affecting the renin–angiotensin–aldosterone system [105].

## 4. The Role of HIF-1α in Vascular Calcification

It is important to note that HIF, as a key regulatory factor, significantly contributes to the progression of VC by regulating the transformation of smooth muscle cells and affecting calcium and phosphorus metabolism.

### 4.1. The Effect of HIF-1α on VSMC Transdifferentiation

**Figure 3:**
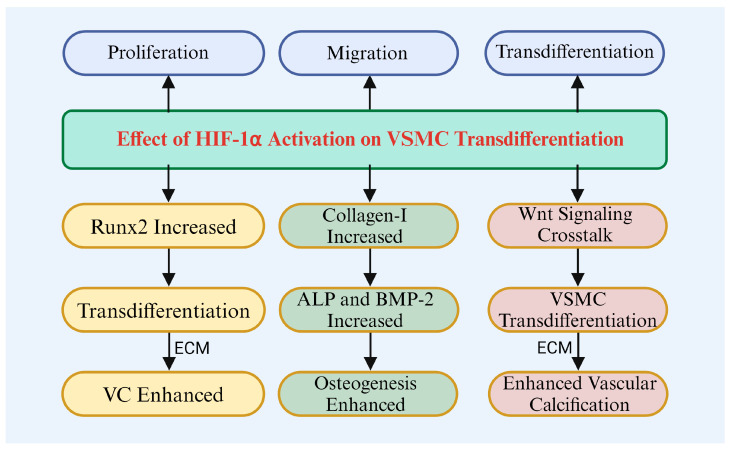
The effect of HIF-1α on VSMC transdifferentiation.

VC is a complex pathological process primarily driven by VSMCs (Figure 3). Under high phosphorus or low oxygen conditions, VSMCs undergo phenotypic changes and acquire osteoblast-like phenotypes. This environment stimulates the expression of HIF, enhances its activity, and induces the transdifferentiation of VSMCs [22,75]. Negri and Mokas et al. suggest that HIF-1α is important in the transdifferentiation of VSMCs, particularly in low-oxygen environments [22,75]. HIF-1α activation not only influences the proliferation and migration of VSMCs but also induces their transformation into osteoblast-like cells, promoting calcification, which is closely linked to the development and progression of cardiovascular diseases [22,75].

Further research has shown that HIF-1α promotes the transformation of VSMCs into osteoblast-like cells by upregulating osteogenic genes, including Runx2, thereby accelerating calcification [3,106]. Lee and Song et al. demonstrated that HIF-1α promotes osteoblast differentiation via Runx2 and significantly upregulates the expression of bone-related genes such as ALP, COL-1, and BMP2 [107,108]. Qi and Chen et al. found that HIF-1α is involved in both osteogenesis and osteoclastogenesis, influencing bone degradation, and its dysfunction is linked to various bone metabolic diseases [109,110].

HIF-1α also promotes the osteogenic transformation of VSMCs by activating Wnt signaling. Majmundar found that HIF-1α regulates Wnt signaling in a context-dependent manner [111]. In stem cells, HIF-1α activates Wnt signaling, whereas in colon cells it exerts inhibitory effects. This suggests that HIF-1α interacts with Wnt signaling through distinct pathways, influencing VSMC transformation and the development of related diseases [111]. Transdifferentiated VSMCs produce increased ECM components, providing structural support and promoting calcium salt deposition, thereby accelerating the process of VC [75].

### 4.2. Regulation of HIF-1α on Calcification-Related Factors

**Figure 4:**
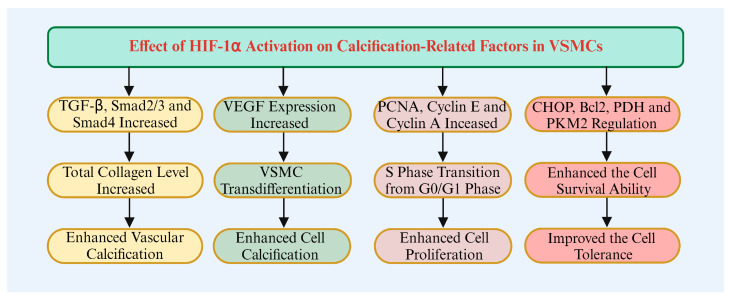
The regulation of HIF-1α on calcification-related factors in VSMCs.

HIF-1α modulates multiple signaling pathways related to calcification by regulating cytokine expression. For example, upregulation of HIF-1α increases TGF-β and VEGF levels, which are critical for cell proliferation and calcification (Figure 4). Mingyuan et al. found that HIF-1α upregulated TGF-β, Smad2/3, p-Smad2/3, Smad4, and total collagen levels in normal and scar tissue fibroblasts. Under hypoxic conditions, HIF-1α activates TGF-β signaling, promoting collagen deposition and calcification [112]. Alique et al. found that HIF-1α is associated with increased VEGF levels in atherosclerotic plaques. In the inflammatory process, HIF-1α activation promotes VEGF expression, thereby promoting calcification [113]. Mokas et al. found that HiPO4 treatment activates HIF-1α and upregulates specific genes, such as VEGF, promoting the osteogenic transformation of VSMCs [75]. Zhao et al. found that HIF-1α promotes new blood vessel formation by regulating VEGF expression. In this process, HIF-1α interacts with TGF-β signaling, affecting hemodynamic changes during calcification and promoting its occurrence [114].

Under hypoxic conditions, HIF-1α regulates cell cycle-related proteins, driving the transition from the G1 to the S phase, thus promoting VSMC proliferation, which is essential for vascular repair and regeneration. Lambert et al. found that HIF-1 inhibitors significantly reduce VSMC proliferation. PCNA and Ki-67 labeling further demonstrated the critical role of HIF-1α in regulating cell proliferation [115]. Huang et al. found that hypoxia promotes PCNA, cyclin E, and cyclin A expression, leading to the transition of VSMCs from the G0/G1 to the S phase. This process is mediated by HIF-1α, underscoring its crucial role in cell cycle progression [116].

Furthermore, HIF-1α inhibits pro-apoptotic protein expression and enhances cell tolerance to hypoxia and other stressors [21]. Mylonis et al. found that HIF-1α inhibitors increase cell tolerance by modulating HIF-1α activity and reducing pro-apoptotic signals [117]. Delbrel et al. found that HIF-1α co-expresses with the endogenous stress marker CHOP, which promotes apoptosis in certain cells. This highlights HIF-1α’s role in regulating cellular stress responses, thus influencing cell survival and tolerance [118]. Zhao et al. found that HIF-1α inhibits tumor cell apoptosis by downregulating Bcl2, an anti-apoptotic protein. This involves the IL-6/STAT3/Bcl2 pathway, which enhances cell survival in hypoxic environments, thus boosting tolerance [119]. Hayashi et al. found that HIF-1α expression inhibits DNA repair mechanisms and induces DNA damage, leading to cellular tolerance to damage [120]. Qannita et al. found that HIF-1α stability is closely linked to glycolysis induction and pyruvate dehydrogenase inhibition, leading to lactate accumulation. In tumor cells, this metabolic shift enhances survival and tolerance in hypoxic conditions [121]. McGettrick et al. found that HIF-1α influences PKM2 function, and inhibiting the PKM2-HIF-1α interaction decreases glycolytic enzyme expression, reducing glycolysis activity. This regulation enhances cell survival and tolerance in hypoxic conditions [122].

Additionally, HIF-1α may promote calcification by downregulating mineralization inhibitors, such as matrix Gla protein [123]. For instance, in a high-phosphorus environment, HIF-1α activation enhances the osteogenic differentiation of valve interstitial cells, linked to reduced expression of mineralization inhibitors [29,75].

### 4.3. Interaction Between HIF-1α and Inflammation in Vascular Calcification

**Figure 5:**
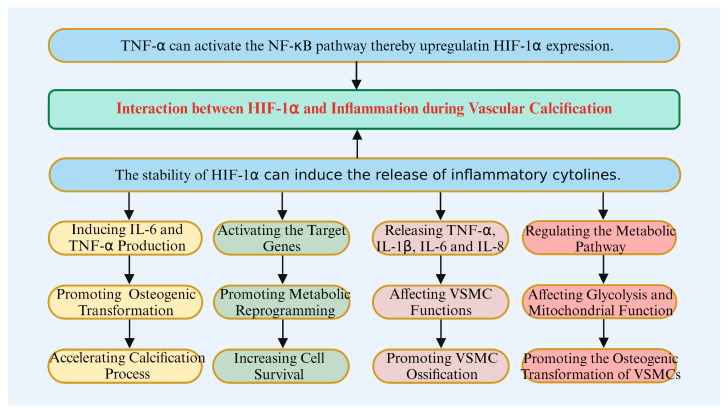
The interaction between HIF-1α and inflammation in vascular calcification.

HIF-1α not only mediates the hypoxic response but also amplifies the inflammatory response by inducing inflammatory factors such as IL-6 and TNF-α [124,125]. These factors further drive the osteogenic transformation of VSMCs, accelerating the calcification process (Figure 5). Kietzmann and Majmundar et al. showed that HIF-1α stability is regulated by oxygen concentration. Under hypoxia, HIF-1α stability increases, activating multiple downstream target genes [126,127], promoting cellular metabolic reprogramming [55], enhancing lactate fermentation and glucose uptake [45], and supporting cell survival under low oxygen conditions [128,129]. Taylor et al. found that the absence of HIF-1α significantly reduces the inflammatory response, especially after infection or injury. HIF-1α enhances the inflammatory response by promoting the release of inflammatory factors [130]. Malkov et al. found that HIF-1α directly regulates the production of pro-inflammatory cytokines, such as TNF-α, IL-1β, IL-6, and IL-8, in synovial fibroblasts of rheumatoid arthritis, highlighting the contribution of HIF-1α to chronic inflammatory diseases [131]. Cui et al. showed that various inflammatory cytokines, including TNF-α and IL-1β, promote VSMC ossification by affecting their function [132]. Song and Wang et al. found that macrophages promote VSMC ossification by releasing inflammatory factors, thereby forming calcified plaques [89,133].

In parallel, inflammatory factors can activate HIF-1α signaling through multiple pathways. For instance, TNF-α activates the NF-κB signaling pathway, thereby upregulating HIF-1α expression [49,134,135,136]. Taylor and Scholz suggested that bacterial products, such as LPS, promote HIF-1α transcription through NF-κB-dependent pathways, potentially enhancing HIF-1α activity by stimulating inflammatory cytokine release [55]. Ruan et al. showed that NF-κB activation not only promotes inflammatory responses but also enhances HIF-1α expression [56].

In an inflammatory environment, the activation of HIF-1α prompts cells to adapt their metabolism and function to the dual stresses of hypoxia and inflammation, potentially leading to the transformation of VSMCs into osteoblast-like cells [3,4]. Negri found that under hypoxic conditions, HIF-1α promotes the transformation of VSMCs into osteoblast-like cells by upregulating osteogenic-related genes [22]. Balogh et al. demonstrated that HIF-1α activation promotes VSMC transformation into osteoblast-like cells and induces intracellular calcium deposition [21]. Yao et al. showed that HIF-1α promotes osteogenic transformation of VSMCs in an inflammatory environment by modulating cellular metabolism, contributing to the development of VC [137]. Chen et al. found that HIF-1α regulates VSMC metabolic pathways in the context of inflammation, promoting their transformation into osteoblast-like cells [110]. Additionally, HIF-1α can promote the osteogenic transformation of VSMCs under both hypoxic and inflammatory conditions by modulating glycolysis and mitochondrial function [22].

Taken together, these studies highlight a strong link between HIF-1α activation and the development of VC.

## 5. Molecular Mechanism of HIF-1α Regulating Vascular Calcification

### 5.1. The Effect of Oxygen-Sensing Mechanisms

**Figure 6:**
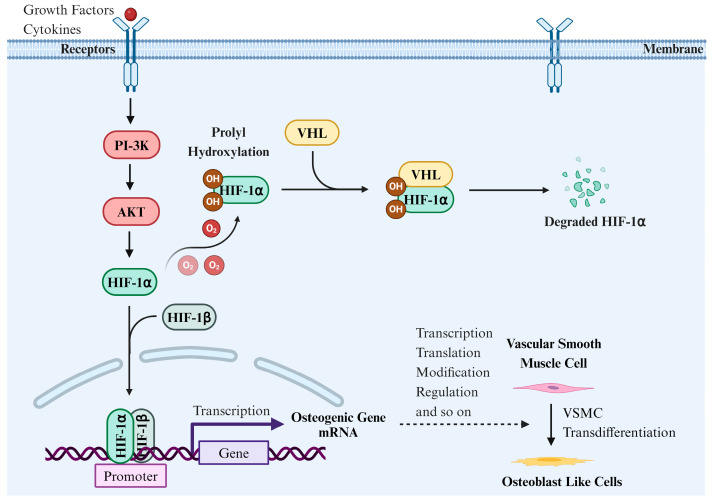
The oxygen-sensing mechanism of HIF-1α regulating vascular calcification.

HIF-1α plays a key role in regulating the differentiation of VSMCs into osteogenic-like cells via oxygen-sensing mechanisms during VC (Figure 6) [2,138,139]. For instance, in a high-phosphorus environment, hypoxic conditions activate the HIF-1α pathway, promoting the osteogenic differentiation of VSMCs and enhancing inorganic phosphorus-induced calcification [75]. Negri and Mokas et al. found that HIF-1α upregulates osteogenic gene expression under hypoxic conditions, promoting the differentiation of VSMCs into osteoblast-like cells, thereby enhancing cell mineralization and osteogenic potential [22,75]. Additionally, HIF-1α can influence the phenotypic transformation of VSMCs by regulating target gene expression. For instance, HIF-1α activation can increase the production of VEGF, which is crucial for vascular remodeling and growth [116].

HIF-1α is an oxygen-sensitive transcription factor, and its stability and activity are tightly regulated by oxygen levels [139]. PHDs are key enzymes responsible for regulating HIF-1α degradation [140,141]. Under normoxic conditions, PHDs use oxygen to hydroxylate proline residues at two specific sites on HIF-1α. The hydroxylated HIF-1α is ubiquitinated and rapidly degraded via the VHL-mediated ubiquitin–proteasome pathway [139]. Weidemann and Johnson found that in the absence of pVHL, the oxygen-dependent stability and activity of HIF-1α were significantly altered, suggesting that the VHL regulatory mechanism on HIF-1α is crucial for responding to changes in oxygen levels [24]. Without VHL, the degradation and stability regulation of HIF-1α is impaired [24]. Thus, under conditions of sufficient oxygen, the level of HIF-1α is typically lower.

Under hypoxic conditions, the activity of PHDs is inhibited, preventing the hydroxylation of HIF-1α, thereby avoiding VHL-mediated degradation and allowing its intracellular accumulation [139]. The accumulated HIF-1α is transferred to the nucleus, where it binds with HIF-1β to form an active transcription complex, triggering the expression of genes related to calcification [2,138,139]. This process involves a decrease in oxygen concentration that inhibits HIF-1α degradation, promotes its accumulation in VSMCs, induces their differentiation into osteoblast-like cells, and accelerates calcification [2,138,139]. For instance, Mokas and Tóth et al. found that targeted inhibition of HIF-1α expression in a mouse model significantly reduced VSMC calcification, highlighting the crucial role of HIF-1α in the calcification process, particularly under hypoxic conditions, where its activation promotes the expression of calcification-related genes [75,142]. Zhou et al. found that HIF-1α regulates the transdifferentiation of VSMCs in high phosphate environments, suggesting that HIF-1α activation not only promotes the transformation of the cell phenotype but also accelerates VC progression [77].

### 5.2. Regulation of HIF-1α on Metabolic Pathways

**Figure 7:**
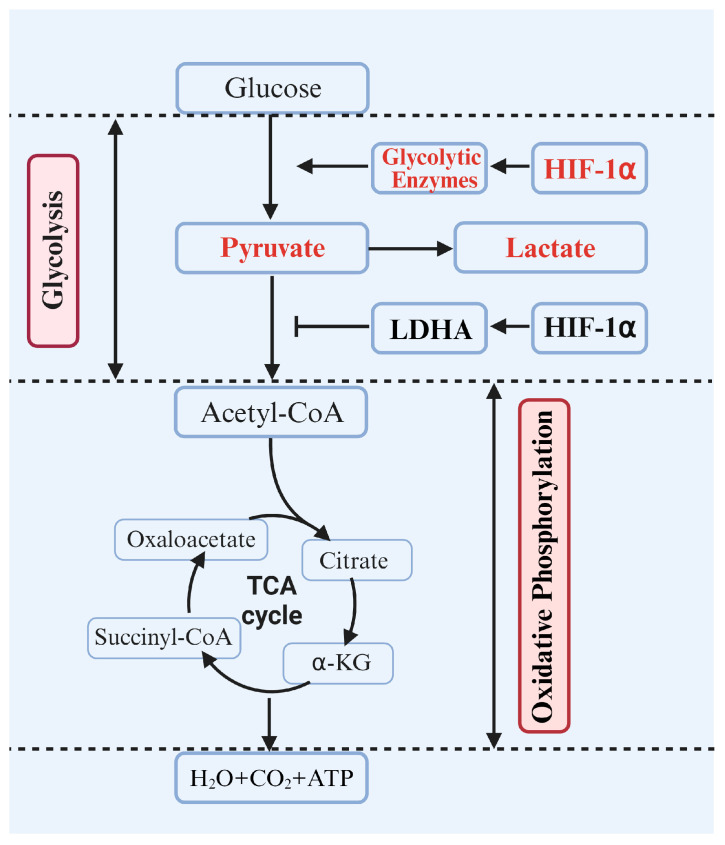
The regulation of metabolic pathways by HIF-1α.

During VC, HIF-1α indirectly affects the functional status of VSMCs by regulating cellular metabolic pathways, particularly glycolysis and oxidative phosphorylation, thereby influencing the calcification process (Figure 7) [143]. For instance, Basheeruddin and Qausain suggested that HIF-1α regulates changes in cellular metabolic pathways, such as glucose utilization and mitochondrial energy production, enabling cells to sustain energy production and reduce oxidative damage. This energy maintenance is crucial for the calcification process [144]. Under hypoxic conditions, HIF-1α stability increases, activating key enzymes in the glycolytic pathway, such as glucokinase, phosphofructokinase, and pyruvate kinase, promoting the conversion of glucose to lactate [145,146,147]. For instance, Kierans and Taylor demonstrated that HIF-1α promotes lactate production by enhancing the expression of LDHA, helping cells maintain energy production and metabolic balance in hypoxic environments [35]. Lee et al. suggested that, under hypoxic conditions, HIF-1α activates genes encoding glucose transporters and glycolytic enzymes, significantly enhancing the glycolytic pathway [25]. Kozlov et al. showed that lactate is not only the end product of glycolysis but also regulates HIF-1α stability, providing feedback to the glycolysis pathway and further promoting lactate production [148]. Carlessi et al. showed that glucagon-like peptide-1 (GLP-1) is an incretin hormone primarily secreted by the intestinal L-cells in response to nutrient intake. When GLP-1 binds to its receptor, it activates HIF-1α signaling, enhances glucokinase activity, and promotes glucose conversion to lactate [149].

Phosphofructokinase 1 (PFK-1) is a key enzyme in the glycolysis pathway, and its activity is regulated by various metabolites. Lu et al. showed that HIF-1α enhances the activity of PFK-1 by activating specific metabolites, thereby accelerating lactate production. When cells experience hypoxia, upregulation of HIF-1α increases the expression of PFK-1, promoting glucose conversion to lactate [150]. Acetoacetate kinase also plays a crucial role in glycolysis. Li and Kim et al. showed that HIF-1α can directly or indirectly regulate the expression of pyruvate kinase, promoting pyruvate production and participating in lactate metabolism [151,152]. Additionally, HIF-1α increases lactate production by activating LDHA, reduces the conversion rate of pyruvate to acetyl-CoA, and thus diverts pyruvate from the TCA cycle [153]. In summary, this process enhances the glycolytic capacity of VSMCs and reduces their dependence on oxidative phosphorylation, leading to significant changes in intracellular energy metabolism.

By favoring the glycolytic pathway, HIF-1α promotes a shift in VSMCs toward anaerobic metabolism, altering the intracellular energy balance and metabolite distribution. For example, in hypoxic environments, HIF-1α enhances cellular adaptability to low oxygen by activating related genes, ensuring a sustained energy supply [25,154]. Simultaneously, activation of HIF-1α leads to significant changes in intracellular metabolic pathways, particularly an increase in the intermediates of glycolysis and the pentose phosphate pathway [155]. Goda and Liang et al. showed that HIF-1α activation alters the distribution of various metabolites, including lactate, pyruvate, and other glycolysis-related intermediates. This change affects not only the metabolic status of cells but may also influence the surrounding microenvironment [156,157]. HIF-1α drives metabolic reprogramming by upregulating glycolysis-related genes [158]. This metabolic reprogramming is closely associated with the transdifferentiation of VSMCs into osteoblast-like cells, thereby promoting VC [2,3].

In addition, HIF-1α downregulates key components of the oxidative phosphorylation pathway [146], inhibiting mitochondrial function and further disrupting cellular energy production and redox balance, thereby exacerbating the pathological process of calcification [21,97,158,159]. Metabolic regulation is a crucial mechanism through which HIF-1α influences VC, serving as a critical link between metabolic reprogramming and the progression of calcification [1,114,160].

### 5.3. Interaction Between HIF-1α and Other Pathways

**Figure 8:**
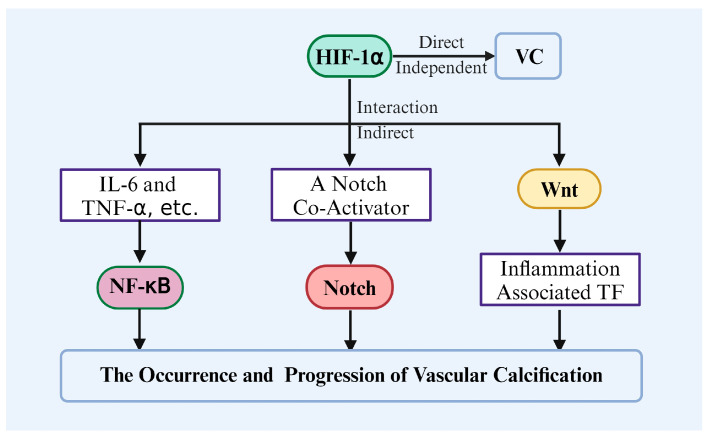
The interaction between HIF-1α and other pathways during the occurrence and progression of vascular calcification.

During the process of VC, HIF-1α not only acts as an independent regulatory factor [21,22,139], but also interacts with other key signaling pathways to form a complex regulatory network (Figure 8). The interaction between HIF-1α and the NF-κB pathway is particularly significant. Under hypoxic conditions, HIF-1α activation can enhance the activity of NF-κB signaling by upregulating inflammatory factors like IL-6/TNF-α, further promoting the osteogenic differentiation of VSMCs and exacerbating calcification. Li and He et al. showed that the activation of HIF-1α in VSMCs and macrophages can trigger the expression of a series of downstream genes, including inflammatory factors IL-6 and TNF-α [96,161]. Among them, IL-6 is a multifunctional cytokine that participates in regulating immune responses and inflammatory processes. Its role in VC is mainly through promoting the osteogenic differentiation of smooth muscle cells, thereby accelerating calcium deposition [96]. TNF-α is also an important pro-inflammatory factor that can activate multiple signaling pathways and promote the calcification process of VSMCs [161]. Escobar Guzman et al. showed that HIF-1α activation upregulates the expression of RANKL, which can further activate the NF-κB pathway by binding to RANK, promoting VC. This indicates the complex interactions between HIF-1α, NF-κB, and RANKL in calcification pathology [162]. Li et al. found that NF-κB not only stabilizes HIF-1α but also participates in regulating multiple aspects of cellular responses, indicating that the feedback mechanism between HIF-1α and NF-κB may exacerbate the occurrence of calcification [163].

In addition, HIF-1α works synergistically with Notch signaling [164,165,166], where the activation of Notch signaling enhances HIF-1α transcriptional activity, jointly regulating the fate determination of VSMCs. Li et al. demonstrated that in neurogenic and acute epilepsy models, HIF-1α directly interacts with the intracellular domain NICD of Notch, thereby enhancing the activation of Notch signaling [167]. Rusanescu and Siebel et al. showed that HIF-1α acts as a co-activator of Notch signaling in various pathological states, directly influencing the transcriptional activity of Notch target genes [168,169]. Kim and Cheng et al. further indicated that HIF-1α, as a global regulatory factor, can modulate Notch signaling, thereby influencing VC under hypoxic conditions [170,171].

In the Wnt/β-catenin pathway, HIF-1α can promote Wnt activation, driving the expression of osteogenic genes and further promoting the calcification process [172,173,174]. Bundy and Li et al. demonstrated that Wnt/β-catenin activation promotes osteoblast differentiation and bone formation, thereby influencing the calcification process [175,176]. Vallée et al. showed that Wnt/β-catenin activation enhances the expression of transcription factors associated with inflammation, exacerbating vascular dysfunction [177]. Additionally, Li and Vallée et al. found that under hypoxic conditions, HIF-1α promotes Wnt/β-catenin activation, influencing bone metabolism and the progression of VC [146,176].

Together, these synergistic effects between signaling pathways position HIF-1α as a multidimensional regulatory center in VC, impacting multiple aspects of pathological processes.

## 6. Potential Therapeutic Target of HIF-1α in Vascular Calcification

**Figure 9:**
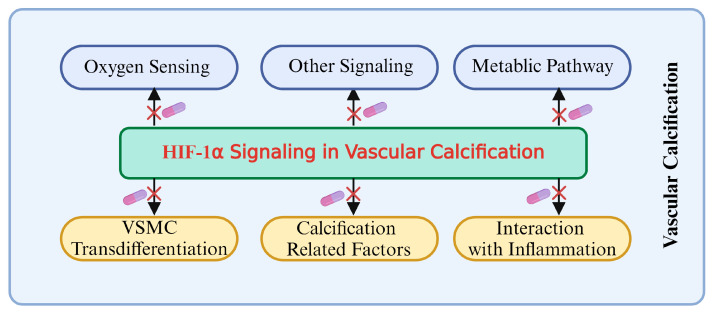
The potential therapeutic target of HIF-1α in vascular calcification.

HIF-1α, as a key regulatory factor in VC, holds significant therapeutic potential (Figure 9) [3,22,58,178]. The stability and activation of HIF-1α under hypoxic conditions are closely linked to the transdifferentiation of VSMCs into osteoblast-like cells, a critical step in VC pathogenesis [2]. Targeting and inhibiting HIF-1α activity or intervening in its downstream signaling pathways can slow or even reverse the osteogenic differentiation of VSMCs, thereby reducing the onset and progression of calcification [22,178]. For instance, oral HIF-1α inhibitors, such as dimethylpentene, have been shown to alleviate VSMC calcification, highlighting the potential of directly targeting HIF-1α for treating calcification-related diseases [22]. Estrogen therapy significantly reduces HIF-1α mRNA/protein levels, subsequently decreasing vascular cell calcification. Estrogen exerts its protective effects by inhibiting HIF-1α expression [22]. Luo et al. demonstrated that capsaicin protects arteries by promoting HIF-1α degradation and inhibiting VSMC calcification [179]. Guo et al. found that inhibiting HIF-1α expression led to a Pit-1 decrease and vice versa. These findings reveal HIF-1α and Pit-1 interaction, suggesting that regulating this pathway may help mitigate VC [180]. Additionally, Tóth et al. found that the activation of the PERK/eIF2a/ATF4/CHOP branch of the endoplasmic reticulum stress response and cooperation between HIF-1a and ATF4 promotes VC induced by a prolyl hydroxylase inhibitor, Daprodustat [181].

In addition, HIF-1α alters the metabolic status of VSMCs by regulating glycolysis and oxidative phosphorylation pathways (Figure 9). Metabolic reprogramming driven by HIF-1α is closely associated with the progression of calcification, suggesting that targeting HIF-1α metabolism offers a novel approach for treating VC [1,2]. For instance, specific inhibition of HIF-1α-mediated glycolysis can restore normal metabolic function in VSMCs, thereby inhibiting their osteogenic transformation and calcification propensity. Balogh et al. demonstrated that the compound chetomin inhibits HIF-1α activity, preventing the effects of hypoxia on osteochondrogenic markers and eliminating ECM calcification [21]. Negri observed that under hypoxic conditions, phosphate-induced VSMC calcification is significantly enhanced, and inhibiting HIF-1α can mitigate this process [22]. Zhu et al. reported that AGEs accelerate VC through the HIF-1α/PDK4 pathway, inhibiting glucose metabolism and suggesting that targeted inhibition of HIF-1α can slow the calcification process [106]. Liu et al. found that the absence of AlkB homolog 1 (ALKBH1), a 2-oxoglutarate and Fe2+-dependent hydroxylase, leads to a decrease in HIF-1α levels, thereby inhibiting glycolysis and related calcification processes [182]. Zhou et al. showed that in a mouse CKD model, the PRMT3 inhibitor SGC707 alleviates VC and inhibits glycolysis, while knockdown of PRMT3 effectively mitigates calcification through the HIF-1α-mediated metabolic pathway [77].

HIF-1α interacts with various signaling pathways, including NF-κB, Notch, and Wnt/β-catenin, all of which play significant roles in regulating VC (Figure 9) [165,166,172]. For instance, Lee et al. demonstrated that under hypoxic conditions, the degradation of IκBα allows NF-κB to translocate into the nucleus, where it interacts with HIF-1α to jointly promote the expression of genes associated with calcification [183]. Recently, therapeutic strategies targeting both HIF-1α and NF-κB have been explored in cancer treatment, showing promise not only in improving treatment efficacy but also in reducing side effects [121,184]. Chang et al. found that mutations in Notch1 resulted in reduced SOX9 expression and the disinhibition of the calcification pathway, suggesting that dysregulation of Notch signaling can promote VC [185]. Landor and Lendahl proposed that developing drugs targeting HIF-1α and Notch could slow the calcification process by inhibiting the expression of calcification-related genes, such as using HIF-1α inhibitors and Notch antagonists [186]. Additionally, Liu and Cong et al. showed that HIF-1α promotes cellular calcification by activating the Wnt/β-catenin pathway, while RTEF-1 can inhibit Wnt/β-catenin signaling to reduce β-glycerophosphate-induced calcification and osteogenic differentiation of VSMCs [187,188].

Targeting HIF-1α and its associated pathways in combination offers a more effective approach to managing the calcification process. Therefore, HIF-1α is not only a crucial molecular marker for understanding the mechanisms of VC but also a key target for the development of future anti-calcification therapies.

## 7. Summary and Prospects

The contribution and regulation of HIF-1α in VC have gradually become a research hotspot. As a key oxygen-sensing transcription factor, HIF-1α not only directly participates in the calcification process by regulating the osteogenic differentiation of VSMCs but also comprehensively influences the complex pathological process of VC through its effects on glycolysis, oxidative phosphorylation, and interactions with other signaling pathways. Existing research indicates that HIF-1α is a significant driving factor for VC, with its expression and activity being markedly influenced by hypoxic conditions, thereby exacerbating the occurrence of calcification in hypoxic environments.

Future research should further explore the multiple regulatory mechanisms of HIF-1α and its complex interactions with other signaling and metabolic pathways. Given the central role of HIF-1α in VC, targeted strategies against HIF-1α are anticipated to become a promising direction for inhibiting or reversing VC. This may include developing specific inhibitors, regulating downstream effectors, or combining interventions targeting these pathways to provide more precise and effective treatment options.

Together, these advances will not only deepen our understanding of the mechanisms underlying VC but also provide new insights for clinical intervention, thereby improving patient prognosis and reducing the incidence and mortality rates associated with cardiovascular diseases.

## Abbreviations/Nomenclature

AGEs: advanced glycation end products, ALP: alkaline phosphatase, ANGPT1: angiopoietin 1, ANGPT2: angiopoietin 2, bHLH: basic helix loop helix, BMP2: bone morphogenetic protein 2, CHOP: C/EBP homologous protein, CKD: chronic kidney disease, COL-1: collagen 1, ECM: extracellular matrix, HIF-1α: hypoxia-inducible factor-1alpha, LPS: lipopolysaccharides, NF-κB: nuclear factor-κB, PAS: Per RNT Sim, PCNA: proliferating cell nuclear antigen, PDGFB: platelet-derived growth factor B, PHD: prolyl hydroxylase, Pit-1: phosphate transporter 1, PlGF: placental growth factor, ROS: reactive oxygen species, TCA cycle: tricarboxylic acid cycle, TGF-β: transforming growth factor-β, VC: vascular calcification, VEGF: vascular endothelial growth factor, VHL: Von Hippel-Lindau protein, VSMC: vascular smooth muscle cell.
